# Reducing ambulance conveyance for older people with and without dementia: evidence of the role of social care from a regional, year-long service evaluation using retrospective routine data

**DOI:** 10.29045/14784726.2021.12.6.3.58

**Published:** 2021-12-01

**Authors:** Chloe Lofthouse-Jones, Phil King, Helen Pocock, Mary Ramsay, Patryk Jadzinski, Ed England, Sarah Taylor, Julian Cavalier, Carole Fogg

**Affiliations:** South Central Ambulance Service NHS Foundation Trust ORCID iD: https://orcid.org/0000-0001-8118-3934; South Central Ambulance Service NHS Foundation Trust ORCID iD: https://orcid.org/0000-0001-7736-7183; South Central Ambulance Service NHS Foundation Trust; University of Warwick ORCID iD: https://orcid.org/0000-0001-7648-5313; Public contributor; University of Portsmouth; South Central Ambulance Service NHS Foundation Trust ORCID iD: https://orcid.org/0000-0002-6752-0807; South Central Ambulance Service NHS Foundation Trust ORCID iD: https://orcid.org/0000-0002-8009-2843; South Central Ambulance Service NHS Foundation Trust ORCID iD: https://orcid.org/0000-0002-2488-7158; Southern Health NHS Foundation Trust; University of Southampton; South Central Ambulance Service NHS Foundation Trust ORCID iD: https://orcid.org/0000-0002-3000-6185

**Keywords:** dementia, emergency medical services, social care

## Abstract

**Introduction:**

Older people, especially those with dementia, have a high risk of deterioration following admission to hospital. More than 60% of older people attended by South Central Ambulance Service (SCAS) clinicians are conveyed to hospital, although many conveyances may not have been due to life-threatening conditions. We aimed to understand patterns of conveyance and alternative referral pathways used following ambulance attendance to an older person.

**Methods:**

Service evaluation, using routinely collected, anonymised electronic records.

**Participants:**

Electronic records of people aged ≥75 years for whom an ambulance was dispatched between April 2016 and March 2017 within the geographical boundaries of SCAS NHS Foundation Trust, who were alive on arrival of the ambulance. Conveyance rates are described according to patient and emergency-call characteristics. Logistic regression was used to produce adjusted odds ratios for conveyance. Alternative referral pathways used are described.

**Results:**

Of 110,781 patients attended, 64% were conveyed to hospital. Factors associated with reduced odds of conveyance included out-of-hours calls (adjusted odds ratio (aOR) 0.82 [0.79–0.85]), living alone with a care package or with family plus care package (aOR 0.66 [0.62–0.69]; aOR 0.58 [0.54–0.62] respectively) and a record of dementia (0.91 [0.87–0.96]). Living in a nursing home was associated with an increased risk of conveyance (aOR 1.25 [1.15–1.36]). Patients with dementia with more income were significantly less likely to be conveyed than those with less income. Alternative referral services were used in 22% of non-conveyed patients, most commonly GP, out-of-hours and falls services.

**Discussion:**

People aged ≥75 years have high rates of conveyance, which are influenced by factors such as out-of-hours calls, dementia and receipt of social care. Low use of alternative referral services may reflect limited availability or difficulty in access. A better understanding of how these factors influence ambulance clinician decision-making is integral to improvement of outcomes for older people.

## Introduction

The number of older people requiring acute, secondary care services in the UK continues to rise, with an 11.3% increase in emergency department attendances and a 4.8% increase in hospital admissions between 2015 and 2017 (Care Quality Commission, 2018). Dementia is prevalent among older people, affecting one in six people over the age of 80, and it is estimated that over 1 million people will be living with dementia in the UK by 2025 (Alzheimer’s Society, n.d.). Emergency ambulance attendances for older people, particularly those with dementia, are common and often require provision of care not related to injury or urgent clinical need (Buswell et al., 2016; Pocock et al., 2018). Once the ambulance crew have addressed the patient’s immediate medical needs, a decision must be made as to whether the patient will be conveyed to hospital for further care and assessment. However, older people frequently have long hospital stays which reduce independence, and their complex medical and social care needs may result in delayed transfers of care and new care home admissions (Bradshaw et al., 2013; NHS Benchmarking Network, 2014; Timmons et al., 2015). Older patients with cognitive impairments or dementia have an even higher risk of deterioration in a hospital environment, and have higher mortality in hospital or shortly after discharge and more frequent readmissions (Fogg et al., 2018, 2019).

Between 2011/2012 and 2016/2017, the observed 70% increase in potentially avoidable emergency department attendances for people with dementia corresponded to cuts in social care funding (Hutchings et al., 2018). These instances of avoidable admissions may be better managed in a care setting appropriate to patient need, such as systems to support people to maintain their health and well-being at home (Care Quality Commission, 2018; Steventon et al., 2018). It is also suggested that patients with dementia requiring assistance with certain activities of daily living and those with a history of falls have more frequent ambulance attendances and are more likely to be hospitalised (Toot et al., 2013; Voss et al., 2018). Actions to address this may include Computerised Clinical Decision Support – a series of menu-based decisions for call handlers, which has been shown to double patient referrals to falls services, while maintaining patient safety outcomes (Snooks et al., 2014).

A recent audit highlighted that around 60% of older people with dementia attended by the South Central Ambulance Service (SCAS) are conveyed to hospital (Pocock et al., 2018). Emergency ambulance clinicians’ conveyance decisions regarding this patient group may be influenced by the alternative care provision available, which may vary according to times of the day or week (O’Hara et al., 2015). We aimed to describe current care pathways for older people following emergency ambulance attendance and explore the association of patient and call characteristics with hospital conveyance to see if it was possible to identify ways to potentially decrease inappropriate emergency department admissions. We performed a service evaluation to explore associations between conveyance decisions made by ambulance staff for older people and factors such as out-of-hours periods, triage grade, social care provision and presence of dementia, and to describe the alternative referral pathways used during this period.

## Methods

### Objectives

1. To describe conveyance rates for older people with an emergency ambulance attendance.
2. To explore whether conveyance rates differed according to the characteristics of the call or the patient demographic, socioeconomic and clinical characteristics.
3. To describe alternative referral pathways used.

### Design

Service evaluation using routinely collected, anonymised retrospective electronic records.

### Setting

Patients with emergency ambulance attendances within the geographical boundaries of SCAS NHS Foundation Trust, including Hampshire, Berkshire, Buckinghamshire and Oxfordshire, serving more than 4 million people.

### Population

Records from the following attendances were included: (i) patients aged ≥75 years; (ii) an electronic record with date and time of attendance available between 1 April 2016 and 31 March 2017; (iii) attendance by emergency ambulance clinicians, including registered paramedics, nurses or doctors, ambulance technicians and associate ambulance practitioners; (iv) within a Clinical Commissioning Group (CCG) area with ≥70% electronic records available (i.e. reflecting areas staffed mainly by SCAS crews rather than private providers). Records for patients who were known to be deceased at the time of ambulance arrival on scene and visits from Patient Transport Services were excluded.

### Data sources and extraction of dataset

Electronic patient records (EPRs) are created at the scene by emergency ambulance staff using the MobiMed Smart electronic tablet (Ortivus, Sweden). The EPR system is used to collect patient clinical and social history, incident details and clinical information. Data are entered into the EPR via a touchscreen on a tablet at the scene, through menus and interactive, self-expanding boxes as well as sections of free text for additional detail about the examination where necessary. Data are transferred to a warehouse and downloaded daily to the SCAS Business Intelligence Team. The Microsoft SQL Server Management Studio was used to extract data from the SCAS data warehouse. Records with the term ‘dementia’ entered in the EPR were identified using queries of free-text fields, as described previously (Pocock et al., 2018). Additional contextual data from several data sources, such the Clinical Commissioning Group (CCG) area, were accessed via Qlikview. The EPR dataset was anonymised, uploaded into a standalone application and matched with incident numbers using Computer Aided Dispatch (CAD) information to link to the triage grade.

### Data analysis

Descriptive statistics were calculated for the total number of emergency attendances and the proportion of conveyances, and summarised according to: (1) a record of dementia; (2) year quarter (based on 3-month aggregations); (3) time period, that is (i) within GP opening hours, defined as 08:00 to 18:00 Monday to Friday, (ii) weekday ‘out of hours’ (18:00 to 08:00 Monday to Thursday, including 08:00 Friday morning), (iii) weekends (18:00 Friday evening to 08:00 Monday morning); (4) triage grade categorised as: ‘Urgent’ – healthcare professional pre-booked admissions where patient triage has already occurred, ‘Red’ – patient’s condition considered to be life-threatening, ‘Green’ – patient’s condition considered not to be life-threatening (National Audit Office, 2017); (5) care arrangements, categorised as: (i) living alone, (ii) living alone with a care package, (iii) with family/carer for dependent, (iii) with family/carer for dependent and care package, (iv) residential care, (v) nursing care (if two or more categories were ticked, the record was categorised as the one with the highest care needs); (6) measures of deprivation: (i) index of multiple deprivation (IMD) rank, and (ii) Income Deprivation Affecting Older People Index (IDAOPI) rank, matched through the Lower Super Output Area (LSOA) on the patient’s home postcode, but if missing (n = 5431), matched to an ‘on-scene’ postcode where available, and categorised by quintiles. Univariable logistic regression was used to identify which factors were associated with conveyance. Categories for out-of-hours weekdays and weekends were combined due to similar results. A forward step-wise multivariable regression model was used to identify factors most relevant for consideration in adaptation of future care. Variables were retained in the model if both the Akaike and Bayesian Information Criterion (AIC/BIC) decreased following their introduction. As people with dementia have specific guidelines regarding conveyance which may influence conveyancing patterns (National Institute of Clinical Excellence, 2018), multivariable analyses were also stratified by presence of the term ‘dementia’ in the EPR. Analyses were performed using Stata version 15.1 (College Station, Texas).

### Patient and public involvement

The need for this work was identified by the SCAS Patient Forum, who were concerned that sometimes their family, friends or neighbours were taken to hospital due to a lack of alternative care and that, once in hospital, they took a long time to return home. A Patient Public Involvement group based in a large acute hospital in the region highlighted difficulties in knowing how to access appropriate care for increasing care needs and wanted to know how the current organisation of care impacts on people’s conveyance to hospital, and what could be done to improve the situation. The results of the evaluation will be shared with these groups, who will assist with dissemination to a public audience, and help to define next steps in the work.

## Results

### Characteristics of emergency attendances and patients

The dataset comprised 110,781 ambulance attendances to people aged ≥75 years during one year (88.7% of total calls to this age group in the region). 64.1% (n = 71,052) of patients were conveyed to hospital. 16.5% (n = 18,288) of the sample had a record of ‘dementia’ in one or more of the free-text fields in the EPR and 83.5% did not. Ambulance attendance and patient characteristics are presented in Table 1. The conveyance rate in patients with a record of ‘dementia’ was 59.0%, as compared to 65.2% in those without.

**Table 1.** Characteristics of ambulance attendances and conveyancing decisions for the total population and stratified by a record of ‘dementia’.

	All patients aged 75+ N = 110,781				Patients with a record of ‘dementia’ N = 18,288		Patients with no record of ‘dementia’ N = 92,493	
	Total		Total conveyed: N = 71,052		Total conveyed: N = 10,796		Total conveyed: N = 60,256	
	n	%^a^	n	%^b^	n	%^b^	n	%^b^
**Characteristics of attendance**								
**Quarter**								
April–June 2004	27,043	24.4	17,256	63.8	2535	58.6	14,721	64.8
July–Sept 2004	26,118	23.6	16,502	63.2	2443	57.7	14,059	64.3
Oct–Dec 2004	28,022	25.3	18,064	64.5	2832	59.7	15,232	65.4
Jan–March 2005	29,600	25.7	19,230	65.0	2986	59.9	16,244	66.0
**Day of the week**								
Monday	16,017	14.5	10,549	65.9	1532	60.5	9017	66.9
Tuesday	15,260	13.8	9,783	64.1	1486	59.1	8297	65.1
Wednesday	15,463	14.0	10,047	65.0	1468	57.6	8579	66.4
Thursday	15,367	14.1	10,206	65.3	1510	60.7	8696	66.1
Friday	16,389	14.8	10,766	65.7	1557	60.2	9209	66.7
Saturday	16,354	14.8	10,056	61.5	1689	58.5	8367	62.1
Sunday	15,661	14.1	9,645	61.6	1554	56.9	8091	62.6
**Time category**								
In hours	46,532	42.0	32,325	69.5	4636	64.1	27,689	70.5
Out-of-hours (weekdays)	25,399	22.9	15,023	59.2	2338	54.0	12,685	60.2
Weekends	38,850	35.1	23,704	61.0	3822	56.9	19,882	61.9
**Triage grade**								
Green	55,064	50.0^c^	28,302	51.4	4908	47.9	23,394	52.2
Red	42,770	38.8	30,200	70.6	4055	66.0	26,145	71.4
Urgent response	12,411	11.3	12,169	98.1	1775	97.5	10,394	98.2
Missing	536	–	381	71.1	58	74.4	323	70.5
**Characteristics of patient**								
**Age band**								
75–79	23,806	21.5	16,160	67.9	1417	60.3	14,743	68.7
80–84	30,004	27.1	19,564	65.2	2587	59.2	16,977	66.2
85–89	30,592	27.6	19,523	63.8	3561	60.0	15,962	64.7
90+	26,379	23.8	15,805	59.9	3231	57.3	12,574	60.6
**Sex**								
Male	46,200	41.9^c^	30,611	66.3	4119	60.5	26,492	67.3
Female	64,122	58.1	40,193	62.7	6645	58.2	33,548	63.7
Not recorded	459	–	248	54.0	32	52.5	216	54.3
**Chief complaint**								
Falls	28,920	26.5^c^	13,837	47.9	2,994	46.9	10,843	48.1
Cardiovascular/cardiac arrest	9512	8.71	8,168	85.9	593	76.6	7,575	86.7
Injury, accident	7054	6.46	4,802	68.1	865	63.6	3937	69.1
General medical	60,242	55.2	42,349	70.3	5986	68.6	36,363	70.6
Mental health	631	0.58	219	34.7	76	45.5	143	30.8
Social	2841	2.60	593	20.9	150	22.9	443	20.3
Not recorded	1581	–	1084	68.6	132	60.8	952	69.8
**Care/living arrangements**								
Alone	24,268	29.0^c^	16,082	66.3	887	60.7	15,195	66.6
Alone plus care package	11,283	13.5	5727	50.8	1056	49.4	4671	51.1
With family	31,578	37.7	21,766	68.9	1941	59.2	19,825	70.1
With family plus care package	5159	6.16	2693	52.2	696	49.6	1997	53.2
Residential care	7127	8.51	4629	65.0	2266	62.9	2363	67.1
Nursing home	4306	5.14	3171	73.6	1431	70.5	1740	76.5
Not recorded	27,060	–	16,984	62.8	2519	57.6	14,465	63.8
**Index of multiple deprivation (IMD) quintile**								
Quintile 1 (most deprived)	7,629	7.03^c^	4961	65.0	728	62.5	4233	65.5
Quintile 2	14,000	12.9	8887	63.5	1342	60.1	7545	64.1
Quintile 3	18,682	17.2	11,803	63.3	1929	58.3	9901	64.4
Quintile 4	26,075	24.0	16,945	65.0	2506	59.7	14,439	66.0
Quintile 5 (least deprived)	42,128	38.8	27,005	64.1	4011	58.2	22,994	65.3
Unavailable	2,267	–	1424	62.8	280	57.5	1144	64.3
**Income Deprivation Affecting Older People Index (IDAOPI) quintile**								
Quintile 1 (most deprived)	6639	6.12^c^	4274	64.4	582	62.7	3692	64.7
Quintile 2	16,034	14.8	10,137	63.2	1695	61.0	8442	63.7
Quintile 3	20,574	19.0	13,081	63.6	1921	57.6	11,160	64.7
Quintile 4	25,028	23.1	16,183	64.7	2477	58.9	13,706	65.8
Quintile 5 (least deprived)	40,239	37.1	25,953	64.2	3841	58.6	22,112	65.7
Unavailable	2267	–	1424	62.8	280	57.5	1144	64.3

^a^ Column percentages, i.e. proportion of characteristic in the category, divided by total population.

^b^ Row percentages, i.e. proportion of patients within each category characteristic who were conveyed, with the denominator for each column given in the header.

^c^ Percentages calculated using the denominator of records with known values.

Attendances were more frequent between October and March, with higher conveyance rates in the same period. Mondays, Fridays and Saturdays were the busiest days for attendances, with highest conveyance rates on Mondays and Fridays. 58% of attendances were during out-of-hours periods (including weekends), and 50% were lower-category emergency calls (‘green’ triage category). Overall, the conveyance rate reduced across age bands, from 67.9% in patients aged 75–79 to 59.9% in patients aged ≥90, although there was less of a trend in patients with a record of ‘dementia’. Ambulance attendances to female patients were more frequent (58.1% females vs 41.9% males), although conveyance rates were lower (62.7% females vs 66.3% males), and there was less difference in conveyance rates between the sexes in patients with a record of ‘dementia’ (58.2% females vs 60.5% males). Around a quarter of all attendances had a fall recorded as the chief complaint, including mechanical (39.5%), fall from standing (23.0%) and unexplained (16.4%). Almost half the patients with falls were conveyed. Living/care arrangements were recorded for 75.6% of the cohort, with 42.5% of attendances to patients living alone, 43.9% living with family and 14% living in nursing/residential homes. Conveyance rates for patients with a record of ‘dementia’ were lower than those with no record across all characteristics.

### Characteristics associated with conveyance

Most factors included in the service evaluation were associated with conveyance in the univariable analysis (Table 2). Characteristics significantly associated with reduced conveyance after adjustment included: out-of-hours calls; age groups 80–84 and 90 and above; being female; a record of ‘dementia’ in the EPR and a main complaint of a fall; a general medical complaint or mental health or social reasons (as compared to ‘injury/accident’). The presence of a care package for people living alone significantly decreased the risk of conveyance (odds ratio (OR) 0.66 [0.62 to 0.69]) and living with family and a care package decreased the conveyance risk still further (OR 0.58 [0.54 to 0.62]). While those in residential care had a similar risk of conveyance to those living alone, people in nursing homes were more likely to be conveyed to hospital. There was no strong evidence for an association between deprivation indicators and conveyance in the overall population, although the IMD quintile 3 showed a significant protective association.

**Table 2.** Characteristics associated with conveyance: results from univariable and multivariable regression.

	Univariable Regression			Multivariable Regression		
	Unadjusted odds ratio	95% CI	p-value	Adjusted odds ratio N = 80,908	95% CI	p-value
**Call details**						
**Time category**						
In hours^a^	1			1		
Out-of-hours	0.67	(0.65 to 0.68)	<0.001	0.82	(0.79 to 0.85)	<0.001
**Triage**						
Green^a^	1			1		
Red	2.27	(2.21 to 2.23)	<0.001	1.55	(1.49 to 1.61)	<0.001
Urgent response	47.5	(41.8 to 54.1)	<0.001	40.7	(34.6 to 47.9)	<0.001
**Patient characteristics**						
**Age band**						
75–79^a^	1			1		
80–84	0.89	(0.86 to 0.92)	<0.001	0.95	(0.91 to 0.99)	0.024
85–89	0.83	(0.81 to 0.86)	<0.001	0.97	(0.93 to 1.02)	0.249
90 and above	0.71	(0.68 to 0.73)	<0.001	0.88	(0.84 to 0.90)	<0.001
**Sex**						
Male^a^	1			1		
Female	0.86	(0.83 to 0.88)	<0.001	0.95	(0.92 to 0.98)	0.004
**Care/living arrangements**						
Alone^a^	1			1		
Alone plus care package	0.52	(0.50 to 0.55)	<0.001	0.66	(0.62 to 0.69)	<0.001
With family	1.23	(1.09 to 1.17)	<0.001	1.00	(0.96 to 1.04)	0.986
With family plus care package	0.56	(0.52 to 0.59)	<0.001	0.58	(0.54 to 0.62)	<0.001
Residential care	0.94	(0.89 to 0.997)	0.390	1.00	(0.94 to 1.07)	0.956
Nursing home	1.42	(1.32 to 1.53)	<0.001	1.25	(1.15 to 1.36)	<0.001
**Dementia**						
No^a^	1			1		
Yes	0.77	(0.75 to 0.80)	<0.001	0.91	(0.87 to 0.96)	<0.001
**Complaint**						
Injury, accident^a^	1			1		
Falls	0.43	(0.41 to 0.45)	<0.001	0.51	(0.48 to 0.55)	<0.001
Cardiovascular/cardiac arrest	2.85	(2.64 to 3.08)	<0.001	2.11	(1.92 to 2.32)	<0.001
General medical	1.11	(1.05 to 1.17)	<0.001	0.82	(0.76 to 0.87)	<0.001
Mental health	0.25	(0.21 to 0.30)	<0.001	0.15	(0.12 to 0.19)	<0.001
Social	0.12	(0.11 to 0.14)	<0.001	0.13	(0.11 to 0.15)	<0.001
**Index of multiple deprivation (IMD) quintile**						
Quintile 1 (most deprived)^a^	1			1		
Quintile 2	0.94	(0.88 to 0.99)	0.023	0.94	0.87 to 1.01	0.103
Quintile 3	0.93	(0.88 to 0.98)	0.009	0.93	0.87 to 0.998	0.045
Quintile 4	1.00	(0.95 to 1.05)	0.945	0.98	0.91 to 1.05	0.541
Quintile 5 (least deprived)	0.96	(0.91 to 1.01)	0.121	0.95	0.89 to 1.01	0.110
**Income Deprivation Affecting Older People Index (IDAOPI) quintile**						
Quintile 1 (most deprived)^a^	1			–^b^	–	–
Quintile 2	0.95	(0.90 to 1.01)	0.100	–	–	–
Quintile 3	0.97	(0.91 to 1.02)	0.240	–	–	–
Quintile 4	1.01	(0.96 to 1.07)	0.669	–	–	–
Quintile 5 (least deprived)	1.01	(0.95 to 1.06)	0.850	–	–	–

^a^ Reference category.

^b^ Not included in multivariable model.

When people with and without a record of dementia were considered separately, the factors associated with conveyance differed (Table 3). Factors such as older age, being female and a main complaint in the ‘general medical condition’ category were associated with reduced odds of conveyance in patients without dementia, but no association was seen in patients with dementia. Although there was no association seen with the IMD quintile in both groups, patients with dementia who were in quintile 3 and above (less deprived) quintiles for the income deprivation affecting older people’s index (IDAOPI) were significantly less likely to be conveyed.

**Table 3.** Characteristics associated with conveyance in multivariable regression stratified by a record of ‘dementia’

	Patients with a record of ‘dementia’ N = 13,424			Patients without a record of ‘dementia’ N = 67,484		
	Adjusted odds ratio	95% CI	p-value	Adjusted odds ratio	95% CI	p-value
**Characteristics of call**						
**Time category**						
In hours^a^	1			1		
Out of hours	0.86	0.80–0.93	<0.001	0.81	0.78–0.84	<0.001
**Triage**						
Green^a^	1			1		
Red	1.47	1.34–1.61	<0.001	1.57	1.51–1.63	<0.001
Urgent response	34.0	23.5–49.2	<0.001	42.4	35.4–50.9	<0.001
**Characteristics of patient**						
**Age band**						
75–79^a^	–^b^	–	–	1		
80–84	–	–	–	0.94	0.90–0.99	0.019
85–89	–	–	–	0.97	0.92–1.02	0.178
90 and above	–	–	–	0.87	0.82–0.91	<0.001
**Sex**						
Male^a^	1			1		
Female	0.98	0.91–1.06	0.640	0.95	0.91–0.98	0.002
**Care/living arrangements**						
Alone^a^	1			1		
Alone plus care package	0.71	0.62–0.83	<0.001	0.64	0.61–0.68	<0.001
With family	0.87	0.76–1.01	0.061	1.01	0.97–1.06	0.571
With family plus care package	0.60	0.51–0.71	<0.001	0.57	0.52–0.61	<0.001
Residential care	1.00	0.87–1.15	0.987	0.99	0.91–1.08	0.853
Nursing home	1.25	1.07–1.46	0.006	1.26	1.13–1.40	<0.001
**Complaint**						
Injury, accident^a^	1			1		
Falls	0.59	0.51–0.68	<0.001	0.49	0.46–0.53	<0.001
Cardiovascular/cardiac arrest	1.69	1.31–2.18	<0.001	2.13	1.91–2.36	<0.001
General medical	0.93	0.80–1.09	0.372	0.79	0.73–0.85	<0.001
Mental health	0.32	0.20–0.50	<0.001	0.11	0.85–0.15	<0.001
Social	0.20	0.15–0.26	<0.001	0.12	0.10–0.13	<0.001
**Index of multiple deprivation (IMD) quintile**						
Quintile 1 (most deprived)^a^	–^b^	–	–	1		
Quintile 2	–	–	–	0.95	0.87–1.03	0.191
Quintile 3	–	–	–	0.94	0.87–1.02	0.125
Quintile 4	–	–	–	1.00	0.92–1.08	0.942
Quintile 5 (least deprived)	–	–	–	0.97	0.90–1.04	0.412
**Income Deprivation Affecting Older People Index (IDAOPI) quintile**						
Quintile 1 (most deprived)^a^	1			–^b^	–	–
Quintile 2	0.87	0.71–1.05	0.150	–	–	–
Quintile 3	0.80	0.66–0.97	0.022	–	–	–
Quintile 4	0.77	0.64–0.92	0.005	–	–	–
Quintile 5 (least deprived)	0.81	0.68–0.97	0.024	–	–	–

^a^ Reference category.

^b^ Not included in multivariable model.

### Alternative referrals

Use of an alternative referral service for patients who were not conveyed was 22.2% (Table 4). Referral to the GP was the most commonly used service (10.6%), followed by the out-of-hours service (6.6%). Social services and mental health services were used in <1% of non-conveyed cases, although conveyance rates for these main complaints were also very low. There were no differences in referral types for patients with or without dementia, although those with dementia had a slightly higher referral rate to falls services and social services.

**Table 4.** Alternative referrals recorded in the Electronic Patient Record for patients who were not conveyed.

Referral service	Emergency attendances with referral services recorded: total N = 39,729	Emergency attendances with referral services recorded: patients with a dementia record N = 7492	Emergency attendances with referral services recorded: patients without a dementia record N = 32,237
Patients with at least one referral pathway:	8807, 22.2%	1732, 23.1%	7075, 22.0%
GP	4223, 10.6%	777, 10.4%	3446, 10.7%
Out-of-hours	2607, 6.6%	512, 6.8%	2095, 6.5%
Falls service	2153, 5.4%	495, 6.6%	1658, 5.1%
TIA clinic	78, 0.2%	12, 0.2%	66, 0.2%
Social services	296, 0.8%	82, 1.1%	214, 0.7%
Mental health	41, 0.1%	16, 0.2%	25, 0.1%
District nurse	525, 1.3%	103, 1.4%	422, 1.3%
Other community service	717, 1.8%	141, 1.9%	576, 1.8%

## Discussion

### Principal findings

Overall, almost two-thirds of people aged 75 and above who called an ambulance were conveyed to hospital, of which almost 50% were ‘non-life-threatening’ calls. There was an association between the provision of a care package at home and reduced hospital conveyance, with a reduction in risk of 34% for people living alone plus care package and 42% for those living with family plus care package, compared to those living alone without a package. Significantly fewer patients were conveyed to hospital during out-of-hours periods. Patients with dementia were less likely to be conveyed to hospital regardless of time period, triage grade and care arrangement, and patients with dementia who had higher incomes were less likely to be conveyed to hospital. Alternative referrals were made for a fifth of patients not taken to hospital, with the most common pathways being to a GP, out-of-hours services or falls services.

### Strengths and limitations

This evaluation reflects a large, generalisable region of the UK and includes a substantial dataset covering a calendar year, accounting for seasonal variation. Missing covariate data reflect the ‘real-world’ nature of information collected during front line emergency situations where the data are not being collected for the purposes of research. Nonetheless, such data provide essential information to inform practice or research. The use of the term ‘dementia’ in the EPRs to identify patients with a dementia diagnosis / suspected dementia could lead to misclassification, for example if dementia was recorded on the EPRs in relation to another household member. However, the proportion of records with ‘dementia’ was almost identical to that recorded in a study which hand-searched paper records (Voss et al., 2018). Bias could also be introduced by recording practices, for example an increased tendency to record ‘dementia’ on the EPR for patients who remain at home. Further prospective studies would be useful to support these results, also including patient acuity, for example NEWS2, linked data from secondary care and more definitive recording of living arrangements and care available at home.

### Relation to other studies and implications

Social care, unlike medical care, is means tested, and studies suggest that hospital admissions and readmissions may increase among people unable to access social care, for example care home beds (Spiers et al., 2019; Steventon et al., 2018). Chronic underfunding, a separation of functions and a lack of information sharing have all contributed to a lack of holistic community-based care (Care Quality Commission, 2018). This evaluation found lower hospital conveyance in patients living at home and receiving additional care, highlighting the importance of investing in the provision of social care, and the co-ordination of social and healthcare in the community. This may be contrasted with findings from an ecological study that found no evidence to suggest that reductions in social care funding (and possibly reduced availability of social care) have led to increases in emergency hospital admissions (Seamer et al., 2019). However, our study uses individual-level data and focuses on emergency department attendances as a consequence of an ambulance attendance, suggesting social care is an important factor determining use of acute care services.

The higher risk of conveyance in patients in a nursing home probably reflects the request for an ambulance only when the home reached the limits of care that medically registered staff could provide. Wider implementation of schemes such as the ‘Red Bag’ (an easily accessible single source of information about the patient) in nursing/residential homes may better guide emergency ambulance clinicians decisions and thus reduce conveyance (NHS England, 2018b).

The lower rate of conveyance out of hours could reflect some services being better able to operate during this time, for example GP home visits, or that patients were less willing to be conveyed to hospital. Alternatively, given the publicity regarding increased mortality in hospital at weekends (Freemantle et al., 2015), ambulance clinicians may anticipate fewer in-hospital services and would prefer to maintain patients at home.

Although patients with falls were less likely to be conveyed, still nearly 50% were taken to hospital, in line with a recent UK study (O’Cathain et al., 2018). Service improvement in assessment of older people for frailty and linkage with specialised community frailty teams may decrease conveyance in future. Increased implementation of initiatives such as end-of-life care bundles, Respect and Advance Care Planning (NHS England, 2018a; ReSPECT, n.d.) and improved communication of this documentation with ambulance services should further reduce inappropriate conveyance for older people. Ambulance service-level factors, including attendance by paramedics with extended skills and the perceptions of risk of senior management, are also factors which may have an impact on decisions made (O’Cathain et al., 2018).

People with dementia were less likely to be conveyed at any time of day, in keeping with findings from another recent UK study (Voss et al., 2018). Deprivation has been associated with increased emergency bed use; and lack of money, both in itself and as a means to access support and equipment, is a barrier to achieving what matters to older people with high support needs (Imison et al., 2012; Katz et al., 2011). The increased conveyance with greater income deprivation in people with dementia in this evaluation suggests that staying at home may be precluded if patients or families cannot pay for additional care or home adjustments to ensure safety and well-being in case of acute illness. The lack of association between age and conveyance in patients with dementia suggests that Advance Care Plans may be more likely to be in place across all age groups for patients with dementia than for those without.

### Unanswered questions and future work

In order to inform planning of additional services or care that may safely retain older patients with unforeseen medical issues in the community, we need to understand specifically what it is about social care provision that influences ambulance clinicians’ decisions, and how it influences patients/relatives’ decisions to stay at home. Further evaluation of the appropriateness of lower conveyance rates in out-of-hours periods is essential to inform equity of services and care over a 24-hour, 7-day week. Understanding how the composition of skills and experience in the attending team influences decision-making in older people’s care would also be valuable. The impact of other factors such as frailty assessments on conveyance decisions and referrals also needs to be considered, as well as the communication between emergency staff and Frailty Intervention Teams at Emergency Department ‘front doors’ (Maloney et al., 2017). Greater individual-level knowledge of the complete emergency journey of such older patients across the health and social care pathway, including data linkage to community and secondary care, is needed to more clearly identify the impact of conveyance decisions, and assess the cost-effectiveness of providing supportive services before, rather than after, the event.

## Author contributions

All authors conceptualised the article and designed the service evaluation and analyses. PK and CF performed the analyses. CLJ, CF, HP and PK drafted the article. All authors contributed to the interpretation of the results and revising the article. CF acts as the guarantor for this article.

## Conflict of interest

None declared.

## Ethics

The service evaluation was approved by the SCAS Clinical Review Group in July 2018. All data were anonymised. The large dataset and group sizes used for analysis further precludes unintended identification of any individuals.

## Funding

None.
